# Does split-suckling influence pre- and postweaning pig growth performance and mortality?

**DOI:** 10.1093/tas/txag039

**Published:** 2026-03-27

**Authors:** Mikayla S Spinler, Jason C Woodworth, Mike D Tokach, Robert D Goodband, Joel M DeRouchey, Katelyn N Gaffield, Ashley R Hartman, Amanda Cross, Brady McNeil, Jordan T Gebhardt

**Affiliations:** Department of Animal Sciences and Industry, College of Agriculture, Kansas State University, Manhattan, KS, 66506, United States; Department of Animal Sciences and Industry, College of Agriculture, Kansas State University, Manhattan, KS, 66506, United States; Department of Animal Sciences and Industry, College of Agriculture, Kansas State University, Manhattan, KS, 66506, United States; Department of Animal Sciences and Industry, College of Agriculture, Kansas State University, Manhattan, KS, 66506, United States; Department of Animal Sciences and Industry, College of Agriculture, Kansas State University, Manhattan, KS, 66506, United States; Department of Animal Sciences and Industry, College of Agriculture, Kansas State University, Manhattan, KS, 66506, United States; Pillen Family Farms, Columbus, NE, 68601, United States; Pillen Family Farms, Columbus, NE, 68601, United States; Pillen Family Farms, Columbus, NE, 68601, United States; Department of Diagnostic Medicine/Pathobiology, College of Veterinary Medicine, Kansas State University, Manhattan, KS, 66506, United States

**Keywords:** colostrum, growth performance, lactation, sow, survivability

## Abstract

A total of 1,513 sows and litters, approximately 505 litters per treatment, were used to determine the effects of two split-suckling protocols on litter growth performance and mortality pre- and postweaning compared to no split-suckling. Sows were blocked by parity and allotted to one of three treatments: no split-suckling (control), or one of two split-suckle protocols. The first split-suckle protocol was based on birth order, where the first 8 pigs born were marked at birth, and at the completion of farrowing, were removed from the sow for 45 min. After 45 min, the first 8 born pigs were returned to the sow and the unmarked pigs born later in the birth order were removed from the sow for 45 min. Then, all pigs were returned to the sow. The second split-suckling protocol was based on body weight (BW.) After farrowing was completed, the 8 heaviest pigs in the litter were removed from the sow for 90 min and then returned to the sow, completing the split-suckle treatment. Litters on either split-suckle treatment were split-suckled within 18 h of birth, after the completion of farrowing if litters were born during the day or the following morning for litters born overnight. Cross-fostering occurred after split-suckling within treatment by 24 h after the completion of farrowing. No differences were observed in litter size or pig weights at the time of split-suckling (d 1) or at weaning, and preweaning mortality did not differ between treatments. Preweaning mortality from split-suckle to weaning was also analyzed by birth weight category within treatment, light (≤1.2 kg), medium (1.2 < × ≤ 1.5 kg), and heavy (≥1.5 kg) pigs, and was not affected by treatment. When litters were evaluated by sow parity, timing of split-suckling (day of birth vs. the following morning), or functional teat count, no differences in preweaning mortality were observed among treatments. A subset of pigs was monitored through the nursery and finishing phases. No treatment differences were observed in average daily gain, average daily feed intake, gain-to-feed ratio, or mortality in either phase. Overall, the split-suckling protocols evaluated in this study did not influence pig growth performance or mortality during the pre- or postweaning periods compared to no split-suckling.

## Teaser

Split-suckling by birth order or body weight had no benefit on pre- or postweaning pig growth performance or mortality compared to no split-suckling.

## Introduction

As a result of genetic selection for high producing sows, litter size in the swine industry has increased (Knap et al. 2023). Average born alive in the U.S. has increased from 13.6 in 2019 to 14.0 in 2024 (MetaFarms 2025). However, sow colostrum production is not related to litter size and does not increase as litter size increases (Devillers et al. 2007). This leads to reduced and unequal colostrum intake in large litters due to greater nursing competition (Jenkins et al. 2025) and the sow’s limited colostrum production capacity (Farmer and Quesnel 2009). When pigs consume less than 200 g of colostrum, preweaning mortality was 43.4% compared to just 7.1% when colostrum intake was greater than 200 g (Devillers et al. 2011; Quesnel et al. 2012). Due to the importance of consuming adequate colostrum and its impact on pig survival, it is necessary to explore management practices that reduce variation in colostrum intake.

Split-suckling is a management strategy that attempts to ensure that all pigs within a litter consume adequate colostrum to reduce preweaning mortality. During split-suckling, a portion of piglets in the litter are temporarily removed from the sow to reduce competition at the sow’s underline, giving the remaining pigs less competition to consume colostrum. Typically, the first born (Morton et al. 2019; Vallet 2019) or heaviest pigs (Vandaele et al. 2020; Arnaud et al. 2023a; Romero et al. 2023) are temporarily removed from the sow to prioritize colostrum intake for later born or light weight pigs. Split-suckling protocols vary, and published research does not suggest one best strategy to increase colostrum intake, reduce variation intake colostrum intake, and decrease preweaning mortality. Vallet (2019) observed that split-suckling by removing the first five pigs born for up to 4 h after the birth of the 9^th^ pig in the litter resulted in a tendency for a reduction in preweaning mortality. Preweaning mortality was reduced from 15.1 to 11.7% in litters that were split-suckled compared to litters not split-suckled. However, Arnaud et al. (2023a) evaluated split-suckling based on body weight (**BW**) by removing the 6 heaviest pigs in the litter for 60 min and then removing the same 6 pigs again for 60 min, 90 min later. Split-suckling did not reduce preweaning mortality compared to no split-suckling.

With differences in split-sucking protocols utilized and varying effects on preweaning mortality, further research is needed using a large sample size to understand the potential impact of this practice on pig performance. Therefore, the objective of this experiment was to evaluate the effects of two different split-suckling strategies on pre- and postweaning growth performance and mortality of pigs compared to no split-suckling. We hypothesized that split-suckled litters would have increased colostrum intake for pigs born later in the birth order and light birth weight pigs and therefore better preweaning survival than litters not split-suckled.

## Materials and methods

The Kansas State University Institutional Animal Care and Use Committee approved the protocol used in this experiment, protocol number 4577.2. The study was conducted at a commercial sow farm in eastern Nebraska (Saint Edward, NE, USA). Sows and their litters were housed in individual farrowing stalls measuring 2.1 × 1.5 m. Pigs were provided 2.1 × 0.5 m on one side of the sow and 2.1 × 0.3 m on the other side with a heat lamp above the larger area. Sows were induced to farrow in the morning on d 115 of gestation using prostaglandin F2alpha (lutalyse; Zoetis, Parsippany, NJ, USA). Needle teeth were clipped, and antibiotic (10 mg; Excede, Zoetis, Parsippany, NJ, USA) intramuscular injections were given within 24 h of birth. Tails were docked, pigs were given an intramuscular injection of Fe (200 mg GleptoForte; Ceva Animal Health, LLC, Lenexa, KS, USA), and male pigs were surgically castrated 3 to 4 d after birth. Creep feed was offered to all litters from d 10 post-farrowing until weaning.

### Animals

A total of 1,513 sows (average parity 3.6; Line 241: DNA, Columbus, NE) and litters (Line 241 × 600, DNA) were used. Sows were moved from the gestation facility to the farrowing facility at approximately d 110 of gestation. On d 110 of gestation, sows and their litter were allotted to one of three treatments, a control and two different split-suckling protocols. In the control treatment, litters were not split-suckled. The split-suckling protocols were based on pig birth order or body weight. Split-suckle treatments were applied within 3 h of the end of farrowing if a sow farrowed during the day and within 18 h of the start of farrowing if a sow farrowed overnight. The end of farrowing was defined as when placenta was passed. For litters split-suckled based on birth order, as the sow was farrowing, the first 8 pigs born were marked with livestock marking paint. Farm staff were present to attend sow farrowing from 0500 to 2400 h. Upon the completion of farrowing, the first 8 pigs were then removed from the sow and placed under a heat lamp in the area adjacent to the sow for 45 min. Pigs were separated from the sow using a plastic board that reached from the front to the end of the farrowing stall (2.1 m) providing an area of approximately 0.58 m^2^. After 45 min, the first 8 pigs born were returned to the sow and the remaining unmarked, later born pigs were removed from the sow and placed behind the board for 45 min. Then, these pigs were placed back on the sow and the split-suckle protocol was complete. If farrowing was not attended to determine pig birth order, the heaviest 8 pigs were removed first, followed by the lighter pigs. For litters that were split-suckled based on body weight, after the completion of farrowing, the 8 heaviest pigs in the litter were removed from the sow and placed under a heat lamp in the stall behind the split-suckle board for 90 min. After 90 min, the 8 heaviest pigs were returned to the sow and the split-suckle protocol was complete.

Functional teat count was recorded at split-suckling (d 1) of the study. Functional teats were defined as an elongated, pointed teat with no visual defects that can produce milk and can be suckled by a pig (Arend et al. 2023). Individual pig weights were taken before split-suckle treatments were applied. Day 1 of the study was the day split-suckling occurred: for sows that farrowed during the day, this was the day of birth, and for sows that farrowed in the late evening or overnight, it was the following morning. Pigs were given an ear tag for individual identification when weights were recorded before split-suckling. After the split-suckle treatment was applied, litters were cross fostered within treatment by 24 h after the completion of farrowing. Fallback pigs were identified between d 2 to 12 post-farrowing as small and gaunt pigs too light to compete with their littermates. These pigs were removed from their sow and placed with a nurse sow not included in the study. When fallback pigs were removed from the sow’s litter and placed onto a nurse sows, the weight of the pig and the date were recorded.

Individual pig weights were recorded again the day before weaning (average d 20) on all pigs. All preweaning mortalities were recorded, including the date and reason for death. At the time of split-suckling, after litter equalization, and at weaning, litter size was recorded. Pig count at split-suckling included only pigs that were alive at the time of ear tagging and weighing. Preweaning mortality only included pigs that were weighed and tagged and does not include pigs that died before initial weights were taken or fallback pigs that were removed from the litter. The 1,513 litters included in the study all contained 10 pigs or greater.

On a subset of approximately 45 litters per treatment, blood samples were taken from every pig in the litter. Blood samples were collected approximately 24 h after the birth of the first pig in the litter. Three mL of blood were collected from the jugular vein using a non-heparinized blood collection tube (Monoject, Covidien, Minneapolis, MN, USA) and a 20 G × 13 mm needle (Neogen, Lansing, MI, USA). At the time of blood collection, the pig’s weight was also recorded. Serum was separated from whole blood through centrifugation at 1,800 × g for 30 min and stored at −80°C until analysis for immunocrit ratio. Briefly, 50 μL of serum was mixed with a 40% ammonium sulfate solution and centrifuged (12,000 × g) in a hematocrit centrifuge tube (Fisher Scientific, Waltham, MA, USA) for 10 min. Immunocrit ratio was calculated by taking the ratio of the precipitate to the length of the serum tube (Vallet et al. 2013).

Additional analysis was conducted to identify if there was a subset of the population where split-suckling positively affected growth performance or reduced preweaning mortality. Additional analyses included determining if there were differences in split-suckle treatment based on litter size, teat count, timing of split-suckle application, or sow parity. Preweaning growth performance and mortality were analyzed on a subset of sows where litter size at split-suckling was greater than functional teat count (*n* = 729). Functional teat count categories were: 14 or less (*n* = 729) or 15 or more (*n* = 676). Timing of split-suckling was divided into two categories: split-suckling immediately after farrowing (*n* = 784) or the following morning for litters that farrowed overnight (*n* = 223). Sows were divided into four parity groups: parity 1 (*n* = 339), parity 2 (*n* = 293), parity 3 to 4 (*n* = 426), and parity 5 and greater (*n* = 455).

A subset of pigs, 2,208 pigs split equally across the three treatments (32 pens per treatment) were followed into the nursery to measure postweaning growth performance and mortality. Pigs that were followed into the nursery were selected because they were born the week of intensive blood collection to measure immunocrit ratio. Nursery pens were 1.8 × 3.0 m with feeder space taking up 0.31 m^2^, allowing for 0.23 m^2^ per pig. Pigs were penned by treatment and fed the same diets in 3 phases. Feed intake was measured on a pen basis using a computerized feeding system (Gestal Evo feeding system, Jyga Technologies, St-Lambert-de-Lauzon, QC, CA). Individual pig weights were taken at the end of the nursery period (48 to 52 d postweaning) before pigs were moved to a finishing facility.

Of the 2,208 pigs that were followed into the nursery, a subset of 882 pigs were followed into the finishing facility (14 pens per treatment). Pigs that had blood samples collected preweaning were selected to be followed into the finisher. During the finisher period, all pigs were fed the same 6 dietary phases. Individual pig feed intake was measured during the finishing period (Nedap Feeders, Nedap, Groenlo, NL). Finisher pens were 2.9 × 5.7 m with 2 computerized feeders (Nedap Feeders, Nedap, Groenlo, NL) in each pen, accounting for 1.2 m^2^ each, allowing for 0.67 m^2^ per pig of floor space. Before the first marketing event, individual pig weights were taken (81 d after transfer from the nursery). Growth performance, including average daily gain (**ADG**), average daily feed intake (**ADFI**), gain: feed ratio (**G: F**), and mortality were determined for the nursery and finishing periods.

### Statistical analysis

Performance data were analyzed using the lmer function of R software, version 1.4.171 as a randomized complete block design. Sow and litter were considered the experimental unit depending on the response variable. Treatment was a fixed effect. Block (sow parity) and farrowing room were considered random effects. Preweaning mortality, percentage of fallback pigs fostered to nurse sows, and litter weight coefficient of variation at split-suckling and weaning were analyzed using a binomial distribution. Preweaning mortality was also analyzed by BW category within litter (light, medium, or heavy) using a binomial distribution. To create the light, medium, and heavy BW categories, the distribution of pig BW at split-suckling was divided into three categories. Litter growth performance and mortality were also analyzed on a subset of the population, where litter size at split-suckling was greater than sow functional teat count. Treatment comparisons were determined considering the interaction of the split-suckle treatment and parity as well as the interaction between split-suckle treatment and timing of split-suckling and sow functional teat count. For the analysis of immunocrit ratio, treatment was a fixed effect, and sow was considered a random effect. The nursery and finisher data were analyzed using treatment as a fixed effect with nursery or finisher room included in the model as a random effect. Pen was considered the experimental unit. Results are considered significant at *P* ≤ 0.05 and marginally significant at 0.05 < *P* ≤ 0.10.

## Results

### Overall population

As expected, no differences were observed among split-suckle treatments for sow parity, functional teat count, or weaning age (Table 1). There were no differences in litter size at split-suckling, after litters were equalized, or at weaning (d 20). Litter and pig weight at split-suckling and at weaning and preweaning ADG were not different among treatments. Litter weight coefficient of variation did not differ among treatments at the time of split-suckling or at weaning. Preweaning mortality from split-suckling to d 2, d 2 to weaning, and split-suckling to weaning was not affected by treatment. There was no difference in the percentage of fallback pigs that were removed and placed with a nurse sow. Preweaning mortality within BW category, light: ≤ 1.2 kg, medium: 1.2 to 1.5 kg, or heavy: ≥ 1.5 kg, was not different among the three split-suckle treatments.

**Table 1:** The effect of split-suckle protocol on litter and pig growth performance and preweaning mortality^1^.

	Split-suckle protocol^2^				
	Control	Birth order	Birth weight	SEM	*P* =
**No. of sows and litters, n**	506	504	503		
**Parity**	3.6	3.6	3.6	0.88	0.995
**Teat count, n^3^**	14.7	14.5	14.6	0.06	0.121
**Wean age, d**	20.0	20.0	20.0	0.13	0.983
**Litter size, n**					
** Split-suckle**	14.9	15.0	15.0	0.46	0.889
** Equalization**	14.5	14.6	14.6	0.17	0.638
** Weaning**	12.3	12.2	12.4	0.25	0.733
**Litter weight, kg**					
** Split-suckle**	20.2	20.3	20.4	0.99	0.704
** Weaning**	67.9	67.6	68.4	2.78	0.536
**Mean pig BW^4^, kg**					
** Split-suckle**	1.36	1.36	1.37	0.039	0.849
** Weaning**	5.51	5.53	5.52	0.161	0.843
**Pig ADG, g/d**	219	220	219	7.0	0.837
**Litter coefficient of variation, %**					
** Split-suckle**	17.2	19.3	18.8	1.65	0.302
** Weaning**	14.8	14.6	14.8	0.65	0.965
**Preweaning mortality, %**					
** Split-suckle to d 2**	2.5	2.0	2.2	0.37	0.178
** Split-suckle to weaning**	10.6	10.6	10.4	0.75	0.922
**Fallback pigs %^5^**	6.1	7.1	6.5	1.02	0.111
**Preweaning mortality by BW^4^ category, %**					
** Split-suckle to weaning**					
** Light, ≤ 1.2 kg**	15.7	17.3	16.3	1.30	0.411
** Medium, 1.2 < × ≤ 1.5 kg**	8.8	8.4	8.7	0.68	0.871
** Heavy, ≥ 1.5 kg**	7.2	6.4	6.6	0.56	0.550

1 A total of 1,513 mixed-parity sows (Line 241, DNA, Columbus NE) and litters were used from birth until weaning.

2 Sows and their litter were allotted to one of three treatments. Control litters were not split-suckled. In the split-suckling treatment based on birth order, the first eight pigs born were removed from the sow for 45 min then placed back with the sow and the remaining pigs were removed for 45 min. In the split-suckling treatment based on birth weight, the eight heaviest piglets were removed from the sow for 90 min before being returned.

3 Teat count includes only functional teats.

4 BW- body weight.

5 Fallback pigs unable to compete with littermates for milk consumption were removed from the litter and placed on nurse sows.

For pigs sampled for immunocrit ratio, there were no differences in pig BW or weight gain at any time point in the study (Table 2). No differences were observed among the three treatments for immunocrit ratio, indicating similar colostrum intake among pigs.

**Table 2:** The effect of split-suckle protocol on pig weight gain and immunocrit ratio^1^.

		Split-suckle protocol^2^			
	Control	Birth order	Control	SEM	*P* =
**No. of sows and litters, n**	45	45	44		
**Parity**	3.6	3.8	3.6	0.33	0.862
**Mean pig BW^3^, kg**					
** Split-suckle**	1.32	1.38	1.37	0.027	0.330
** d 2**	1.38	1.42	1.41	0.031	0.636
** Weaning**	5.28	5.17	5.34	0.086	0.358
**Pig gain, g**					
** Split-suckle to d 2**	56	40	43	9.4	0.440
** Split-suckle to weaning**	3,928	3,769	3,947	79.1	0.212
**Immunocrit ratio^4^**	0.076	0.079	0.078	0.0028	0.784

1 Blood samples were collected on 134 whole litters to determine immunocrit ratio 24 h after the birth of the first pig in the litter. At the time of blood collection, pig weight was also recorded.

2 Sows and their litter were allotted to one of three treatments. Control litters were not split-suckled. In the split-suckling treatment based on birth order, the first eight pigs born were removed from the sow for 45 min then placed back with the sow and the remaining pigs were removed for 45 min. In the split-suckling treatment based on birth weight, the eight heaviest piglets were removed from the sow for 90 min before being returned.

3 BW- body weight.

4 A 3 mL blood sample was taken from each pig on approximately 45 litters per treatment. Serum was pulled from whole blood and used to determine immunocrit ratio.

### Litter size at split-suckling greater than functional teat count

A subset of the population was analyzed to determine the effect of split-suckling when litter size was greater than functional teat count (237 to 250 sows per treatment; Table 3). Within this subset, there were no differences in parity or functional teat count, litter size at split-suckling, after litter equalization, or weaning. No differences were observed for litter weight or pig BW at split-suckling or weaning or pig ADG. Preweaning mortality from split-suckling to d 2 was greater (*P* < 0.05) in litters not split-suckled compared to litters split-suckled based on birth order, with litters split-suckled based on body weight intermediate. However, preweaning mortality from split-suckling to weaning, as well as the percentage of fallback pigs placed with nurse sows were not different among treatments.

**Table 3:** The effect of split-suckle protocol on litter and pig growth performance and preweaning mortality for sows that had greater pig count than functional teat count at split-suckle^1^.

		Split-suckle protocol^2^			
	Control	Birth order	Birth weight	SEM	*P* =
**No. of sows and litters, n**	237	252	240		
**Parity**	4.0	3.8	3.9	0.87	0.999
**Teat count, n^3^**	14.4	14.2	14.2	0.07	0.092
**Litter size, n**					
** Split-suckle**	16.8	16.8	17.0	0.32	0.829
** Equalization**	15.0	15.1	15.3	0.26	0.716
** Weaning**	12.3	12.3	12.4	0.28	0.902
**Litter weight, kg**					
** Split-suckle**	22.2	22.0	22.5	0.76	0.219
** Weaning**	67.8	68.0	68.9	2.81	0.553
**Mean pig BW^4^, kg**					
** Split-suckle**	1.32	1.32	1.33	0.029	0.490
** Weaning**	5.47	5.47	5.49	0.159	0.853
**Pig ADG^5^, g/d**	206	208	208	6.80	0.745
**Preweaning mortality, %**					
** Split-suckle to d 2**	3.3^a^	2.4^b^	2.5^ab^	0.47	0.025
** Split-suckle to weaning**	12.4	11.7	11.6	0.93	0.659
**Fallback pigs, %^6^**	7.9	9.0	8.4	1.17	0.371

^**a–c**^Means in the same row that do not have a common superscript differ (*P* < 0.05).

1 A total of 729 mixed-parity sows (Line 241, DNA, Columbus NE) and litters were used from birth until weaning.

2 Sows and their litter were allotted to one of three treatments. Control litters were not split-suckled. In the split-suckling treatment based on birth order, the first eight pigs born were removed from the sow for 45 min then placed back with the sow and the remaining pigs were removed for 45 min. In the split-suckling treatment based on birth weight, the eight heaviest piglets were removed from the sow for 90 min before being returned.

3 Teat count includes only functional teats.

4 BW- body weight.

5 ADG- average daily gain.

6 Fallback pigs unable to compete with littermates for milk consumption were removed from the litter and placed on nurse sows.

### Interactions between treatment and functional teat count

Sows were divided into two functional teat count categories, sows that had 14 or less functional teats and sows that had 15 or more functional teats (Table 4). No treatment differences nor interactions between split-suckle treatment and functional teat count were observed. Pigs weaned from sows with 15 or more functional teats had a lower (*P* < 0.05) BW at weaning and preweaning ADG compared to pigs weaned from sows with 14 or less functional teats. However, no differences in litter weight at weaning were observed.

**Table 4:** Interactive means between teat count and split-suckle protocol on litter and pig growth performance and preweaning mortality^1^.

			Split-suckle protocol^2^				*P* =			
	Control		Birth order		Birth weight					
Teat Count^3^:	≤ 14	≥ 15	≤ 14	≥ 15	≤ 14	≥ 15	SEM	Treatment × teat count	Treatment	Teat count
**No. of sows and litters, n**	250	236	283	196	267	217				
**Parity**	3.6	3.3	3.6	3.3	3.5	3.4	0.17	0.563	0.912	0.108
**Teat count, n^3^**	13.8	15.6	13.8	15.6	13.7	15.6	0.04	0.855	0.837	0.000
**Litter size, n**										
** Split-suckle**	14.9	15.1	15.1	15.1	15.2	14.9	0.26	0.663	0.673	0.718
** Equalization**	14.5	14.4	14.6	14.6	14.7	14.7	0.27	0.991	0.827	0.844
** Wean**	12.1	12.3	12.1	12.2	12.2	12.5	0.25	0.922	0.943	0.467
**Litter weight, kg**										
** Split-suckle**	20.2	20.3	20.2	20.6	20.6	20.1	1.02	0.143	0.324	0.662
** Weaning**	68.1	67.2	67.6	67.5	68.4	68.1	2.88	0.864	0.686	0.396
**Mean pig BW^4^, kg**										
** Split-suckle**	1.37	1.36	1.35	1.37	1.37	1.36	0.042	0.343	0.311	0.625
** Weaning**	5.58	5.42	5.55	5.47	5.54	5.45	0.169	0.567	0.733	0.003
**Pig ADG^5^, g/d**	222	214	221	217	219	216	7.2	0.425	0.645	0.003
**Preweaning mortality, %**										
** Split-suckle to d 2**	2.7	2.3	2.1	1.9	2.2	2.1	0.42	0.709	0.219	0.327
** Split-suckle to weaning**	10.8	10.5	11.4	9.5	10.7	9.7	0.90	0.455	0.647	0.658
**Fallback pigs, %^6^**	6.8	5.5	7.1	7.0	6.8	6.3	0.86	0.313	0.928	0.044

1 A total of 1,513 mixed-parity sows (Line 241, DNA, Columbus NE) and litters were used from birth until weaning.

2 Sows and their litter were allotted to one of three treatments. Control litters were not split-suckled. In the split-suckling treatment based on birth order, the first eight pigs born were removed from the sow for 45 min then placed back with the sow and the remaining pigs were removed for 45 min. In the split-suckling treatment based on birth weight, the eight heaviest piglets were removed from the sow for 90 min before being returned.

3 Teat count includes only functional teats.

4 BW- body weight.

5 ADG- average daily gain.

6 Fallback pigs unable to compete with littermates for milk consumption, were removed from the litter and placed on nurse sows.

### Interactions between treatment and timing of split-suckling

The timing of split-suckling was analyzed by comparing litters that were split-suckled the same day they were born to those that were split-suckled the day after farrowing (Table 5). This analysis does not include litters on the control treatment that were not split-suckled. There were no interactions observed between treatment and split-suckle timing. A main effect of split-suckle time was observed for pig ADG, where litters split-suckled directly after the completion of farrowing had greater (*P* < 0.05) ADG compared to litters that were split-suckled the morning after birth (litters born in the evening or overnight).

**Table 5:** Interactive means between split-suckle time and split-suckle protocol on litter and pig growth performance and preweaning mortality^1^.

	Split-suckle protocol^2^				*P* =			
	Birth order		Birth weight					
Split-suckle time^3^:	Same day	Next day	Same day	Next day	SEM	Treatment × split-suckle time	Treatment	Split-suckle time
**No. of sows and litters, n**	404	100	380	123	—	—	—	—
**Parity**	3.6	3.6	3.6	3.6	0.88	0.854	0.921	0.933
**Teat count, n^4^**	14.6	14.5	14.6	14.5	0.38	0.801	0.836	0.924
**Litter size, n**								
** Split-suckle**	15.0	15.0	15.2	14.9	0.55	0.652	0.769	0.999
** Equalization**	14.5	14.8	14.6	14.9	0.35	0.971	0.728	0.532
** Weaning**	12.3	12.2	12.4	12.4	0.37	0.864	0.452	0.765
**Litter weight, kg**								
** Split-suckle**	20.2	20.5	20.2	21.1	1.06	0.217	0.770	0.433
** Weaning**	67.9	67.1	68.4	68.8	3.02	0.461	0.533	0.478
**Mean pig BW^5^, kg**								
** Split-suckle**	1.36	1.38	1.36	1.39	0.043	0.479	0.840	0.284
** Weaning**	5.53	5.49	5.51	5.54	0.167	0.237	0.327	0.325
**Pig ADG^6^, g/d**	222	212	220	215	7.0	0.290	0.264	0.002
**Preweaning mortality, %**								
** Split-suckle to weaning**	10.8	9.4	10.6	9.2	1.00	0.965	0.725	0.141
**Fallback pigs, %^7^**	7.2	7.5	6.8	6.8	1.04	0.725	0.439	0.624

1 A total of 1,007 mixed-parity sows (Line 241, DNA, Columbus NE) and litters were used from birth until weaning.

2 Sows and their litter were allotted to one of three treatments. In the split-suckling treatment based on birth order, the first eight pigs born were removed from the sow for 45 min then placed back with the sow and the remaining pigs were removed for 45 min. In the split-suckling treatment based on birth weight, the eight heaviest piglets were removed from the sow for 90 min before being returned.

3 Split-suckle time refers to if a litter was split-suckled the same day they were born or the following morning (litters born overnight).

4 Teat count includes only functional teats.

5 BW- body weight.

6 ADG- average daily gain.

7 Fallback pigs unable to compete with littermates for milk consumption, were removed from the litter and placed on nurse sows.

### Interactions between treatment and parity

Sow parity was divided into four groups: parity 1, 2, 3 to 4, and parity 5 + sows (Supplemental Table 1). There were no interactions between treatment and parity for any response criteria except for the percentage of fallback pigs removed and placed with nurse sows (*P* < 0.05; data not shown). In parity 1 sows, litters not split-suckled had a lower (*P* < 0.05) percentage of fallback pigs compared to litters split-suckling by birth order or body weight. However, in parity 2, 3 to 4, and 5 + sows, the percentage of fallback pigs was not different among treatments.

### Interaction between treatment and BW on immunocrit ratio

The interaction between treatment and piglet BW at split-suckling was evaluated to determine whether immunocrit ratio differed by treatment across BW categories (Table 6). Immunocrit ratio was not affected by treatment split-suckling BW, or their interaction, indicating that immunocrit ratio was consistent across all weight groups. Pigs weighing ≥ 1.5 kg at split-suckling were heavier at all time points and gained more preweaning than pigs weighing < 1.5 kg (*P* < 0.05). Pigs weighing 1.2 to 1.5 kg at split-suckling were also heavier and gained more at all time points than pigs weighing < 1.2 kg at split-suckling.

**Table 6:** Interactive means between split-suckle pig weight and split-suckle protocol on immunocrit ratio and pig gain^1^.

				Split-suckle protocol											
	Control^2^			Birth order			Body weight				*P* =				
BW:	< 1.2 kg	1.2 < × ≤ 1.5 kg	≥ 1.5 kg	< 1.2 kg	1.2 < × ≤ 1.5 kg	≥ 1.5 kg		< 1.2 kg	1.2 < ×≤ 1.5 kg	≥ 1.5 kg		SEM	Treat-ment × BW	Treatment	BW
**Piglets, n**	228	249	176	198	210	217		210	203	229					
**Parity**	3.6	3.6	3.7	3.9	3.8	3.8		3.6	3.6	3.6		0.34	0.999	0.860	0.985
**Immunocrit ratio^3^**	0.073	0.078	0.078	0.076	0.082	0.078		0.077	0.079	0.078		0.0035	0.863	0.636	0.163
**Mean pig BW^4^, kg**															
** Split-suckle**	1.05	1.34	1.65	1.05	1.34	1.68		1.04	1.35	1.67		0.011	0.293	0.829	<0.001
** d 2**	1.10	1.40	1.69	1.09	1.40	1.70		1.10	1.40	1.70		0.017	0.959	0.922	<0.000
** Wean**	4.66	5.21	6.00	4.49	5.09	5.72		4.63	5.36	5.87		0.100	0.207	0.437	<0.001
**Pig gain, g**															
** Split-suckle to d 2**	53	63	49	42	57	24		53	48	30		11.7	0.739	0.354	0.522
** Split-suckle to weaning**	3,591	3,870	4,351	3,416	3,743	4,041		3,550	4,004	4,204		98.4	0.166	0.410	<0.001

1 Blood samples were collected on 134 whole litters, approximately 45 litter per treatment to determine immunocrit ratio 24 h after the birth of the first pig in the litter.

2 Sows and their litter were allotted to one of three treatments. Control litters were not split-suckled. In the split-suckling treatment based on birth order, the first eight pigs born were removed from the sow for 45 min then placed back with the sow and the remaining pigs were removed for 45 min. In the split-suckling treatment based on birth weight, the eight heaviest piglets were removed from the sow for 90 min before being returned.

3 A 3 mL blood sample was taken from each pig on approximately 45 litter per treatment. Serum was separated from whole blood and used to determine immunocrit ratio.

4 BW- body weight.

### Postweaning pig performance

A subset of 2,208 pigs, represented equally across the three treatments, were followed into the nursery (Table 7). Starting BW in the nursery was greater for pigs split-suckled by BW compared to pigs not split-suckled, with pigs split-suckled by birth order intermediate. However, because there was no difference in BW at weaning for the overall data when including all pig weights at weaning, this difference was likely due to chance. Final BW at the end of the nursery (d 48 to 52 postweaning) was not different among treatments. No differences in ADG, ADFI, G: F, or mortality were observed during the nursery period. A subset of 882 pigs that were followed into the nursery were also followed into the finisher phase. No differences in starting or final BW before the first marketing event (d 81 post nursery) were found. No differences were observed in growth or mortality.

**Table 7:** The effect of split-suckle protocol on nursery and finisher pig performance^1^.

		Split-suckle protocol			
	Control^1^	Birth order	Body weight	SEM	*P* =
**Nursery^2^**					
** Pens, n**	32	32	32	—	—
** Starting BW^3^, kg**	5.1^b^	5.1^ab^	5.3^a^	0.05	0.020
** Final BW^3^ (49 d postweaning), kg**	25.2	25.0	25.1	0.29	0.839
** ADG^4^, g**	400	400	394	6.1	0.700
** ADFI^5^, g**	513	517	516	9.1	0.940
** G: F^6^**	0.78	0.78	0.77	0.010	0.549
** Mortality, %**	4.5	3.1	4.1	0.008	0.400
**Finisher^7^**					
** Pens, n**	14	14	14	—	—
** Starting BW^3^, kg**	25.5	24.8	27.8	0.54	0.137
** Final BW^3^ (77 d post-nursery), kg**	102.5	102.9	104.3	1.09	0.332
** ADG^4^, kg**	0.97	0.97	1.00	0.021	0.148
** ADFI^5^, kg**	2.41	2.39	2.44	0.030	0.316
** G: F^6^**	0.40	0.41	0.41	0.008	0.576
** Mortality, %**	3.7	4.1	1.7	0.012	0.200
** Removals, %**	2.7	3.4	2.7	1.057	0.800
** Mortality + removals, %**	6.4	7.7	4.5	1.824	0.300

1 Sows and their litter were allotted to one of three treatments. Control litters were not split-suckled. In the split-suckling treatment based on birth order, the first eight pigs born were removed from the sow for 45 min then placed back with the sow and the remaining pigs were removed for 45 min. In the split-suckling treatment based on birth weight, the eight heaviest piglets were removed from the sow for 90 min before being returned.

2 A subset of pigs, 2,208, were used to evaluate nursery growth performance and mortality.

3 BW- body weight.

4 ADG- average daily gain.

5 ADFI- average daily feed intake.

6 G: F- gain to feed ratio.

7 A subset of pigs, 882, were used to evaluate finishing growth performance and mortality.

## Discussion

One of the primary components of colostrum is immunoglobulins (Verstegen et al. 1998). Immunoglobulins are proteins that provide passive immunity from the sow to the new born pig, but are not able to pass through the sow’s placenta (Dividich et al. 2005). Immunoglobulin concentrations in colostrum rapidly decrease after farrowing, with immunoglobulin G levels being approximately 52 mg/mL in colostrum and 10 mg/mL in milk at 24 h post farrowing (Loisel et al. 2013).

Split-suckling is a strategy that attempts to ensure that all pigs within a litter, especially in large litters, consume adequate colostrum (Arnaud et al. 2023b). Split-suckling strategies reported in the literature aim to ensure adequate colostrum intake, particularly for light birth weight pigs or those born later in the birth order. (Arnaud et al. 2023b). Colostrum quality declines rapidly as time from the start of parturition increases (Farmer 2015). Thus, pigs born later in the birth order receive lower quality colostrum as well as reduced time to consume colostrum compared to pigs born earlier in the birth order. The split-suckle treatment based on birth order was designed to attempt to ensure adequate colostrum intake for pigs born later in the litter by reducing competition.

Multiple split-suckle protocols are used in commercial swine production and data does not indicate one best protocol that leads to reduced preweaning mortality. This variability is reflected when comparing the results of the current study to previous studies. Vallet (2019) observed a tendency for a reduction in preweaning mortality, 15.1 vs 11.7%, when litters were split-suckled by removing the first five pigs born for up to 4 h after the birth of the 9^th^ pig in the litter, whereas the present study found no differences. Vallet (2019) visually assessed pigs via stomach palpation to confirm nursing before removal from the sow. Results suggest that ensuring pigs have nursed before split-suckling may be important, though standardizing on-farm visual assessments for colostrum intake is challenging. In agreement with our results, Morton et al. (2019) observed that split-suckling by birth order resulted in no differences in preweaning mortality or growth performance when split-suckling by removing pigs born in the first half of the birth order for 90 min. Because birth weight influences colostrum intake, split-suckling can also be performed based on pig weight

Devillers et al. (2007) observed that colostrum intake of newborn pigs is correlated with birth weight, with heavier pigs having increased colostrum consumption. Therefore, the split-suckle treatment based on BW was designed to ensure adequate colostrum intake for light weight pigs by reducing nursing competition. For the split-suckle treatment based on BW, the heaviest 8 pigs were removed from the sow for 90 min because this allowed the lighter pigs remaining on the sow to nurse for at least two nursing cycles. Colostrum flow is continuous until 10 to 11 h after the first pig is born, after that, sows let down colostrum every 40 to 50 min (Lewis and Hurnik 1985). Huser et al. (2015) observed that removing the largest pigs in the litter for 2 h increased survival of small pigs weighing less than 850 g by 13% compared to no split-suckling or a rotational protocol where the litter was divided into two groups and alternately removed for 1 h over a 4 h period. However, no differences in pig survivability were observed for pigs weighing greater than 850 g. The study herein did not find any differences among treatments for preweaning mortality among light pigs ≤ 1.2 kg. Studies by Huser et al. (2015) and Vallet (2019) observed improvements in preweaning mortality when pigs were removed for 2 h or longer, which contrasts with the current results, where pigs were removed for either 45 min or 90 min based on split-suckle treatment. In agreement with Donovan and Dritz (2000), Morton et al. (2019), Vandaele et al. (2020), Arnaud et al. (2023a), and Romero et al. (2023), no differences in preweaning mortality were observed when split sucking based on BW.

Vandaele et al. (2020) and Romero et al. (2023) observed that split-suckling by BW reduced pig preweaning growth rate. Romero et al. (2023) observed a reduction in ADG of 20 g/d when pigs were split-suckled based on BW. The split-suckling protocol used by Romero et al. (2023) involved removing pigs with a birth weight greater than 950 g from the sow for 60 min. These pigs were removed in two groups, with each group being removed for 60 min, two times. Vandaele et al. (2020) split-suckled by removing the heaviest pigs from the first half of the birth order for 3 h, followed by the heaviest from the second half of the birth order for 3 h, once daily for the first 3 d after birth. This resulted in decreased preweaning growth rate during the first 3 d after farrowing compared to no split-suckling or a single split-suckling event performed only on the day of birth. The Vandaele et al. (2020) study indicates that split-suckling for multiple days may hurt growth performance of pigs removed from the sow. Arnaud et al. (2023a) and Morton et al. (2019) observed that split-suckling based on BW did not improve pig growth performance or preweaning mortality. Arnaud et al. (2023a) evaluated the effects of split sucking by removing the six heaviest pigs in the litter for 60 min and then removing the same six pigs for 60 min again 90 min later, while Morton et al. (2019) removed the six heaviest pigs in the litter for 90 min one time. The literature indicates that split-suckling based on birth order or BW results in variable responses, with the majority of studies finding no differences compared to no split-suckling.

Devillers et al. (2004) observed a linear relationship between colostrum intake and pig BW gain during the first 24 h after farrowing. Romero et al. (2023) observed that split-suckling increased gain within the first 24 h after birth. However, Vandaele et al. (2020) observed that split-suckling resulted in lower pig gain during the first 24 h after birth. In contrast, the current study observed no differences in gain within the first 24 h after birth, which is in agreement with the results of Morton et al. (2019).

In addition to growth performance, several studies also assessed colostrum intake using immunocrit ratio as an indicator. Immunocrit ratio is a measure of pig serum immunoglobulin level and is used as an indicator of colostrum intake of the pig (Vallet et al. 2013). A higher immunocrit ratio indicates a higher colostrum intake. Similar to the current study, Morton et al. (2019) and Huser et al. (2015) found no differences in immunocrit ratio between pigs split-suckled and not split-suckled. Vandaele et al. (2020) and Arnaud et al. (2023a) estimated colostrum intake using equations developed by Theil et al. (2014) and found no differences between split-suckling and no spilt suckling. Vallet (2019) found that split-suckling by removing the first 5 pigs born for 4 h increased immunocrit ratio in pigs weighing less than 1.0 kg but not in pigs weighing more than 1.0 kg. In the current study, split-suckling did not increase immunocrit ratio in light birth weight pigs weighing less than 1.2 kg. There was no interaction between treatment and BW category for immunocrit ratio, indicating that split-suckling by BW did not affect colostrum intake of lighter weight pigs that remained on the sow during split-suckling based on BW. Similar to the present study, Morton et al. (2019) also evaluated pig birth weights in three categories, light, < 1.11 kg, medium, 1.11 < × ≤ 1.45 kg, and heavy, ≥ 1.5 kg, and found no interaction between treatment and BW category for immunocrit ratio. The lack of difference in immunocrit ratio and pig gain within the first 24 h after birth in the current study indicates that the split-suckle protocols applied in this study did not have an effect on colostrum intake of pigs.

Because of potential confounding variables in split-suckling protocols in the literature (parity, teat count relative to litter size, timing of split-suckling), additional analysis was conducted to determine if split-suckling had a positive effect on subsets of the population. For litters where pig count at split-suckling was greater than functional teat count, split-suckling by birth order resulted in reduced preweaning mortality from split-suckling to d 2 compared to no split-suckling. The average litter size in this subset of the population was 16.9 vs 15.0 in the whole study population, which indicates that split-suckling may be more effective in large litters. However, no significant differences in mortality were observed from split-suckling to weaning, but litters not split-suckled had numerically higher mortality. When looking at the reason for mortality in this population from split-suckling to d 2, litters not split-suckled had a higher percentage of pigs laid on by the sow compared to litters split-suckled by birth order. Donovan and Dritz (2000) evaluated two split-suckling strategies. In the first strategy, the heaviest 50% of the litter were removed for 2 h. In the second strategy, the heaviest 50% were removed for 2 h and then the lightest 50% were removed for an additional 2 h. The split-suckling strategies evaluated were effective in reducing variation in pig ADG, but only in litters with more than nine pigs. This reduction in variation contributed to more uniform pigs at weaning. Collectively, these findings suggest that the greatest potential to gain a benefit from split-suckling is in larger litters. However, in our study, split-suckling did not significantly influence mortality between split-suckling and weaning.

The interaction between split-suckle treatment and parity was assessed because primiparous sows produce less colostrum than multiparous sows (Devillers et al. 2007; Ferrari et al. 2014). Thus, split-suckling may be more effective in gilt litters compared to litters from multiparous sows due to the lower gilt colostrum production. Romero et al. (2023) observed that split-suckling was more effective in gilt versus multiparous sow litters, with pigs from gilts that were split-suckled having greater gain within the first 24 h after birth compared to litters from multiparous sows that were split-suckled. However, no other treatment by parity interactions were observed in the study (Romero et al. 2023). In agreement with the results of the study herein, Romero et al. (2023) observed that split-suckling was not more effective in litters born from primiparous vs multiparous sows for overall preweaning ADG or mortality.

Because colostrum quality changes once farrowing begins, an analysis between split-suckling performed immediately after farrowing versus the following morning for litters that farrowed overnight was conducted. No differences in pre-wean mortality were observed between litters split-suckled directly after the completion of farrowing versus those split-suckled the following morning for litters that farrowed overnight. Pigs split-suckled directly after the completion of farrowing had a greater preweaning ADG compared to litters from sows that farrowed in the evening or overnight that were split-suckled the following morning after birth. The reason for this is that pigs that were split-suckled the next morning had more time to consume colostrum, making them heavier at the time of split-suckling compared to those weighed and split-suckled immediately after birth, which led to a lower ADG. It is important to note that all pigs were split-suckled within 18 h of the birth of the first pig, within the typical time of colostrum production (Quesnel et al. 2012). Due to the induction protocol used on farm, the majority of litters were split-suckled the day of birth (784 litters the same day of birth vs 223 litters the following morning).

Wiegert and Knauer (2019) observed that sow colostrum production and pig colostrum consumption increased as functional teat count increased. Due to the potential effect of functional teat count on colostrum production, the effect of functional teat count was analyzed, and there was no interaction between teat count and treatment. This suggests that split-suckling does not result in a positive effect when functional teat count is below 14 teats vs above 15 teats. Pigs weaned from sows with 14 or less functional teats had greater preweaning ADG and weaning weight compared to pigs weaned from sows with 15 or more functional teats. This is likely because sows with 15 or more teats had numerically greater litter size at weaning. Overall, treatment responses were not affected by the interaction between treatment and parity, spilt suckle timing, or teat count, indicating that the effectiveness of split-suckling is not dependent on these factors.

Colostrum intake has shown long-term effects on pig growth performance, with pigs consuming greater than 290 g of colostrum gaining 2 kg more by 6 weeks of age compared to pigs consuming less than 290 g of colostrum (Devillers et al. 2011). Declerck et al. (2016) observed that as total colostrum intake increased from 0 to 899 g, preweaning and nursery mortality decreased, illustrating the long-term effects of colostrum intake. The greatest improvement occurred when total intake rose from 0 to 228 g, with more modest reductions observed as colostrum intake increased above 228 g. In their study, there also was a positive correlation between colostrum intake and pig weight at weaning and the end of the nursery and finishing period. Due to the long-term impact of colostrum intake, postweaning growth performance and mortality were evaluated.

Only Arnaud et al. (2023a) and Romero et al. (2023) have evaluated the effects of split-suckling on postweaning growth performance. Consistent with the present findings, Romero et al. (2023) observed no effect of split-suckling on postweaning growth compared to no split-suckling. In contrast, Arnaud et al. (2023a) observed a 1.8 kg reduction in market weight in pigs that were split-suckled, attributed to lower weaning weights. Arnaud et al. (2023a) split-suckled by removing the six heaviest pigs for 60 min twice, suggesting that removing pigs multiple times during split-suckling can hurt pig growth performance, which was also observed in the results of the study by Vandaele et al. (2020). However, the current study found that the split-suckling strategies implemented did not affect lifetime growth performance or mortality, likely due to the lack of treatment differences in colostrum intake.

In conclusion, the split-suckling protocols evaluated in this study, split-suckling by birth order or body weight, did not affect preweaning or postweaning growth performance or mortality compared to no split-suckling. The lack of significant differences is likely due to similar colostrum intake across groups, as suggested by pig immunocrit ratios. Findings from this study, along with previous literature, suggest that the effectiveness of split-suckling protocols is variable and split-suckling does not consistently reduce preweaning mortality. Future studies should focus on evaluating other ways to invest time during the early postnatal period to improve pig livability.

## Supplementary Material

txag039_Supplementary_Data

## List of abbreviations

ADFI: average daily feed intake
ADG: average daily gain
BW: body weight
G: F: gain to feed ratio
