# Supplementary material for: Does split-suckling influence pre- and postweaning pig growth performance and mortality?
Source: Transl Anim Sci. 2026 Mar 27;10:txag039. doi: 10.1093/tas/txag039 (PMC13089491; doi:10.1093/tas/txag039)
Supplement: txag039_Supplementary_Data [file txag039_supplementary_data.docx]

| **Supplemental Table 1.** Main effect of sow parity^1^ | | | | | | | | |
| --- | --- | --- | --- | --- | --- | --- | --- | --- |
|  | Parity | | | | |  |  |  |
|  | 1 | 2 | 3 to 4 | 5 + | SEM | | *P* = |  |
| No. of sows and litters, n | 339 | 293 | 426 | 455 |  | |  |  |
| Teat count, n^2^ | 14.6 | 14.6 | 14.6 | 14.5 | 0.05 | | 0.085 |  |
| Wean age, d | 19.9^bc^ | 20.1^ab^ | 20.2^a^ | 19.9^c^ | 0.12 | | 0.002 |  |
| Litter size, n |  |  |  |  |  | |  |  |
| Split-suckle | 13.6^b^ | 15.0^a^ | 15.7^a^ | 15.6^a^ | 0.23 | | 0.001 |  |
| Equalization | 14.3 | 14.8 | 14.8 | 14.7 | 0.22 | | 0.505 |  |
| Weaning | 12.1 | 12.9 | 12.5 | 11.8 | 0.21 | | 0.123 |  |
| Litter weight, kg |  |  |  |  |  | |  |  |
| Split-suckle | 17.5^c^ | 21.6^a^ | 21.8^a^ | 20.2^b^ | 0.20 | | 0.001 |  |
| Weaning | 61.2^c^ | 71.6^a^ | 72.1^a^ | 66.0^b^ | 0.80 | | 0.001 |  |
| Mean pig BW^3^, kg |  |  |  |  |  | |  |  |
| Split-suckle | 1.30^c^ | 1.46^a^ | 1.40^b^ | 1.30^c^ | 0.011 | | 0.001 |  |
| Weaning | 5.07^c^ | 5.64^ab^ | 5.76^a^ | 5.62^b^ | 0.060 | | 0.001 |  |
| Pig ADG^4^, g/d | 200^c^ | 219^b^ | 228^a^ | 228^a^ | 2.3 | | 0.001 |  |
| Preweaning mortality, % |  |  |  |  |  | |  |  |
| Split-suckle to d 2 | 1.9 | 1.6 | 2.8 | 2.8 | 0.23 | | 0.071 |  |
| Split-suckle to weaning | 11.9 | 9.1 | 10.6 | 12.5 | 0.53 | | 0.084 |  |
| Fallback pigs %^5^ | 5.2^c^ | 5.4^bc^ | 6.7^b^ | 9.3^a^ | 0.92 | | 0.001 |  |
| ^1^A total of 1,513 mixed-parity sows (Line 241, DNA, Columbus NE) and litters were used from birth until weaning.  ^2^Teat count includes only functional teats.  ^3^BW- body weight.  ^4^ADG- average daily gain.  ^5^Fallback pigs unable to compete with littermates for milk consumption, were removed from the litter and placed on nurse sows. | | | | | | | | |
